# A 3D Printed Membrane Reactor System for Electrochemical CO_2_ Conversion

**DOI:** 10.3390/membranes13010090

**Published:** 2023-01-10

**Authors:** Andreu Bonet Navarro, Adrianna Nogalska, Ricard Garcia-Valls

**Affiliations:** 1 Department of Chemical Engineering, Universitat Rovira i Virgili, Av. Països Catalans, 26, 43007 Tarragona, Spain; 2 Eurecat, Centre Tecnològic de Catalunya, C/Marcellí Domingo, 2, 43007 Tarragona, Spain

**Keywords:** 3D printed reactor, Nafion membrane, CO_2_ electroreduction, membrane reactor, tin electrocatalyst

## Abstract

Nowadays, CO_2_ electroreduction is gaining special interest as achieving net zero CO_2_ emissions is not going to be enough to avoid or mitigate the negative effects of climate change. However, the cost of CO_2_ electroreduction is still very high because of the low efficiency of conversion (around 20%). Therefore, it is necessary to optimize the reaction conditions. Thus, a miniaturized novel membrane reactor was designed and manufactured in this study, with a shorter distance between the electrodes and a reduced volume, compared with CNC-manufactured reactors, using novel stereolithography-based 3D printing. The reduced distance between the two electrodes reduced the electrical resistance and therefore lowered the overpotential necessary to trigger the reaction from −1.6 V to −1.2 V, increasing the efficiency. In addition, the reduction in the volume of the reactor increased the catalyst area/volume ratio, which also boosted the concentration of the products (from FE 18% to FE 21%), allowing their better identification. Furthermore, the smaller volume and reduced complexity of the reactor also improved the testing capacity and decreased the cost of experimentation. The novel miniaturized reactor can help researchers to perform more experiments in a cost/time-effective way, facilitating the optimization of the reaction conditions.

## 1. Introduction

Atmospheric CO_2_ capture will become mandatory to avoid the future catastrophic effects of climate change. The reason is because even with stopping CO_2_ emissions, the high inertia that global warming has reached will not stop [1]. In previous studies, we developed an efficient CO_2_ capture system that was capable of fixing CO_2_ 10 times more effectively than natural leaves. However, [2,3] the economic cost of absorbing large quantities of atmospheric CO_2_ is still very high. Therefore, the electroreduction of CO_2_ to convert it to products with a higher value is crucial to make this technology competitive on the market [4]. One of the most interesting products that can be obtained from CO_2_ electroreduction is formic acid (FA), due to its ability to store large quantities of electrical energy and to become a future competitor against lithium batteries, with a higher energy density [5]. As described in our previous studies [2,3] using membrane technology, CO_2_ can be trapped by making it react with a sodium hydroxide solution, which converts it into bicarbonate. However, with direct air capture, the saturation of the absorbent solution will take a very long time, and thus the conversion of the CO_2_ in the solution to formic acid will be affected in terms of the reaction’s velocity, as we described previously [6]. Most studies have performed this reaction under ideal conditions in which they pre-saturated the solution by bubbling pure CO_2_ [7], but this does not reflect the real application with atmospheric CO_2_. Consequently, the reaction conditions and parameters must be improved in order to achieve efficient conversion under lower concentrations of CO_2_.

For the electrochemical experiments, bulk tin (Sn) was chosen as the electrocatalyst for CO_2_ electroreduction due to its low cost, ease of use and its selectivity toward FA, as described in previous publications [6]. The reactor’s structure was the same H-type cell as used previously, but with greatly reduced size. Furthermore, a Nafion 117 membrane was used as a proton exchange membrane. The membrane has high proton permeability (H^+^) while maintaining a reasonably low permeability to formic acid [8]. Similar approaches are used for the electrochemical production of methanol [9] and catalytic synthesis [10,11]. The electroreduction of CO_2_/bicarbonate solutions is affected by many parameters, as described in our previous work. In addition, experimentation needs to be carried out by trial and error; therefore, researchers face the challenge of having to perform large numbers of experiments. Thus, the main aim and novelty of this study was designing and manufacturing a reactor as small, simple and easy to handle as possible in order to simplify and increase the speed of testing. In addition, this will also help to reduce the amount of reagents, reducing the economic cost of experiments while making it easy to assemble, disassemble and manipulate. Moreover, by increasing the ratio of the catalyst’s area to the reactor’s volume, the concentration of the products was increased, facilitating their identification and quantification. For that purpose, stereolithography 3D printing tool [12] was used. Nowadays, 3D printing is gaining interest in many research fields [13]. This novel manufacturing technique presents several advantages over conventional subtractive ones [14], e.g., faster prototyping, the possibility of printing more complex objects and therefore reducing the total number of parts, simplifying assembly, and reducing its size and thus the manufacturing cost.

## 2. Materials and Methods

### 2.1. Materials

Tin foil (99.8% trace metals basis; 0.25 mm thick), provided by Alfa Aesar (Haverhill, MA, USA), was used as working electrode together with a platinum wire 37 mm long with a diameter of 0.5 mm, provided by Ossila (Sheffield, England), as the counter-electrode. In addition, a carbon nanotube-based mini-reference electrode from NTSensors S.L. (Tarragona, Spain) was used. Furthermore, highly pure KHCO_3_ (Bio Ultra 99.5%), supplied by Sigma-Aldrich (St. Louis, MO, USA), with a content of iron lower than 5 mg/kg was used for preparation of the electrolyte. Milli-Q water was used to prepare the solutions. The proton exchange membrane was a Nafion^®^ 117 membrane (Fuel Cell Store, College Station, TX, USA). Hydrogen peroxide 30% (*v*/*v*) (Fisher Chemical, Loughborough, UK) and 95–97% H_2_SO_4_ (ACS reagent, Madrid, Spain) were used to prepare cleaning solutions for the Nafion membrane. To prepare a standard for 1 H NMR analysis, 99.8% D_2_O from Acros Organic (Sweden, Switzerland) and 99.7% DMSO (Chromasolv Plus, Seelze, Germany) from were used. Formlabs’ clear resin (SLA 3D printing resin) was used for the reactor printing (Somerville, MA, USA).

### 2.2. Design of 3D Printed Reactor

A miniaturized reactor was designed using FreeCAD (0.20.2) and manufactured with a Form 3 SLA 3D printer from Formlabs, considering the following requirements of the electrochemical reduction system:

- The module should consist of two separated and symmetric compartments, cathodic and anodic, with a Nafion 117 proton exchange membrane assembled in between.
- The module should have one hole to accommodate the counter-electrode on the anodic compartment, and another one in the cathodic compartment for the working electrode.
- Each compartment should have two additional holes for a mini-reference electrode and a pH sensor.
- The reactor should have input holes to inject the reagent and output holes to remove it.
- The compartments also should have two holes for fitting thermometers, one in each compartment.
- The compartments should have enough space to fit a magnet for stirring.
- The reactor should be as small as possibly to reduce the amount of reagent and increase the reagent/catalyst area ratio.

Once the reactor had been designed and printed, electrode holders were also manufactured using SLA 3D printing technology by using Form 3 (Formlabs) with clear resin. The 3D designs made with FreeCAD were sliced using Preform software. After printing the reactor, the uncured resin was removed by washing in isopropanol and post-cured with an UV light lamp.

### 2.3. CO_2_ Electroreduction Tests with the Miniature 3D Printed Membrane Reactor

The results obtained with the novel homemade 3D printed reactor were compared with the ones obtained with a previous CNC-manufactured H-cell made of Teflon [6]. CO_2_/bicarbonate electroreduction tests were performed following the procedure described in the previous study in terms of CV and chronoamperometric measurements. Specifically, a 1.5 M bicarbonate solution was used as the CO_2_ source together with bulk tin (Sn) foil as a catalyst. CV scans ranging from −2.0 to 0.1 V for a total of 5 scans at a 0.1 V scan rate were performed to select the optimal working potential in order to avoid the hydrogen evolution reaction [15]. As a result of this, a potential of −1.2 V was selected for the chronoamperometric measurements. The total chronoamperometry time was 1 h, which was more than enough to observe a significant and quantifiable amount of formate by NMR.

The main differences between the old and the new reactor relied on the volume of electrolyte, which decreased from almost 30 mL to 1.5 mL, reducing the cost of the experiment and increasing the sensitivity. Furthermore, the distance between the counter-electrode and the working electrode was reduced, decreasing the internal resistance and reducing the voltage necessary to achieve a certain level of current density. The membrane’s size decreased from 700 to 144 mm^2^, but the ratio between the membrane’s area and the electrolyte’s volumeincreased strongly because the total volume of the reactor was reduced 20-fold.

## 3. Results

The assembly of the novel 3D printed membrane reactor was described, along with the results obtained from the experimental CO_2_ electroreduction. These results were compared with the experiments performed using the previously reported CNC-manufactured reactor [6].

### 3.1. The 3D Printed CO_2_ Electroreduction Membrane Reactor

**Figure 1:**
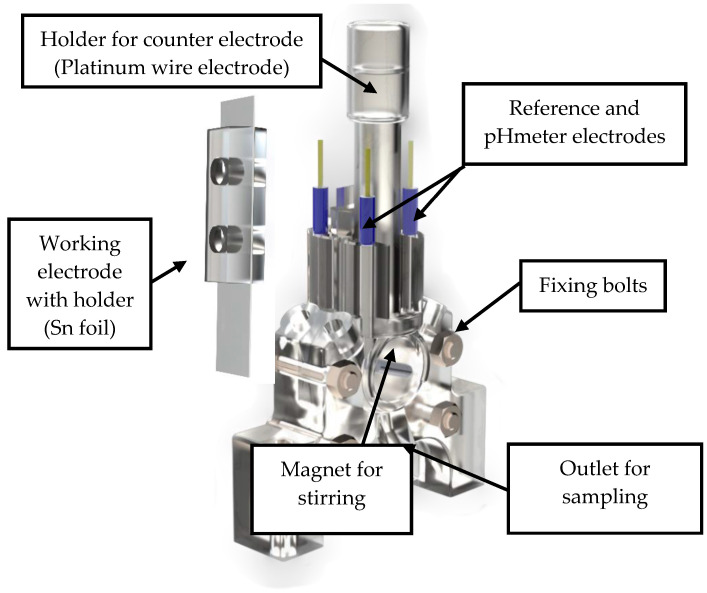
General view of the rendered 3D design of the final prototype of the reactor.

**Figure 2:**
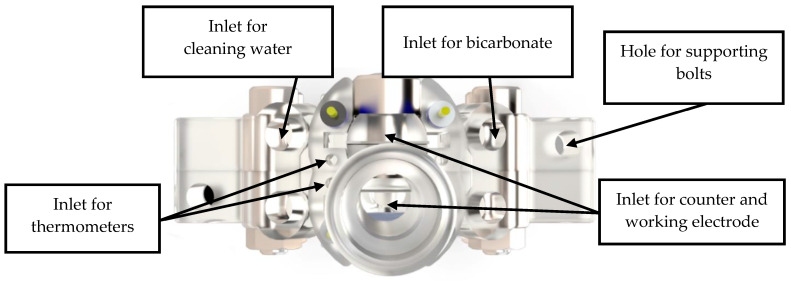
Top view of the rendered design of the final prototype of the reactor.

**Figure 3:**
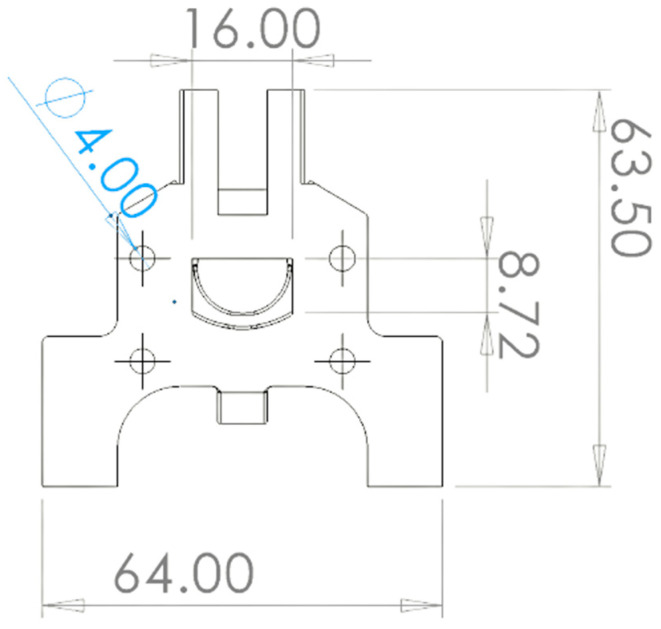
A 2D sketch of the final reactor prototype with its main dimensions in mm.

**Figure 4:**
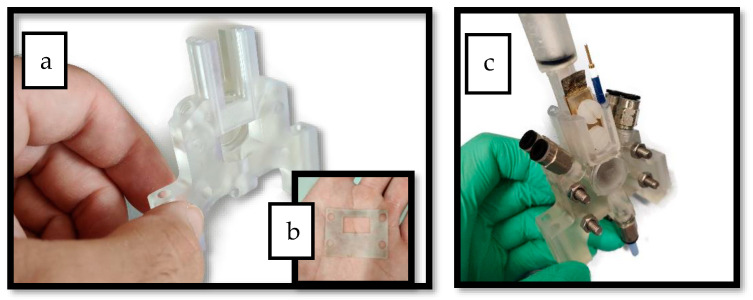
(**a**) Photo of one of the reactor’s compartments after printing. (**b**) Silicon sheet used to seal the gap in between the two compartments. (**c**) Photo of the final assembled reactor after printing.

**Figure 5:**
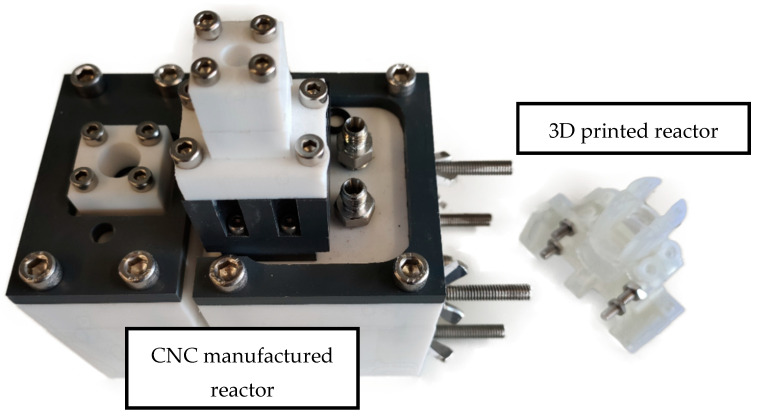
The old CNC manufactured reactor on the left compared with the novel 3D printed reactor on the right.

Figure 1 and Figure 2 show the 3D rendering design obtained with FreeCAD, with all its components, achieved following the guidelines described in Section 2.2. Additionally, Figure 3 shows the dimensions of one of the symmetric compartments. Moreover, a photo of an real 3D printed module is displayed in Figure 4, where it can be observed that both compartments were joined using four bolts with a silicon sealing sheet to avoid any leaking from the gap between them. A Nafion proton exchange membrane (144 mm^2^ and 183 μm thickness) was placed in between the two compartments in order to avoid crossover of the product (FA) to the anodic compartment and thus its re-oxidation. Nafion 117 was chosen due to its high thickness compared with other similar membranes, which resulted in the low permeability of formate [9]. The differences in the size and complexity between the 3D printed reactor and the CNC-manufactured reactor can be observed in Figure 5. All differences between these reactors are summarized in Table 1, where the 20-fold reduction in volume is highlighted, as well as the transparency, easy cleaning, faster manufacturing time, lower manufacturing price, the decrease in weight (almost 47-fold) and the shorter electrode distance (by almost 4 cm). These improvements helped us to increase the testing capacity while reducing the testing costs and increasing the electrical efficiency because of the reduced distance between the electrodes.

### 3.2. Bicarbonate Electroreduction Results

**Figure 6:**
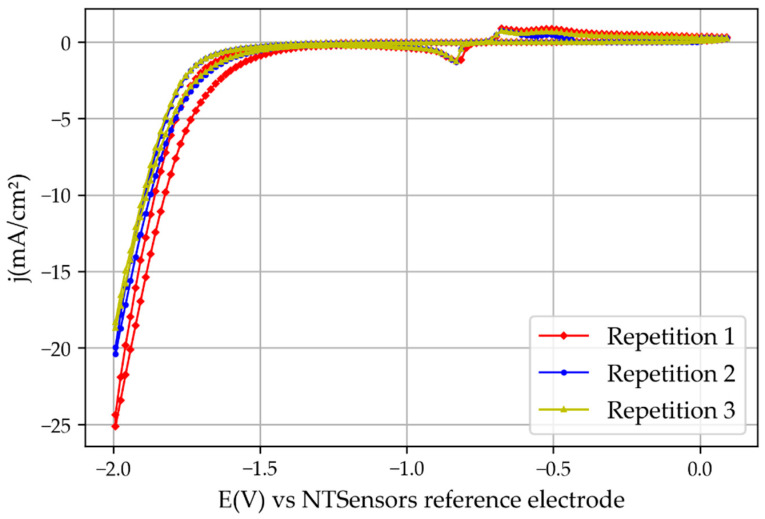
Cyclic voltammetry results obtained with the 3D printed reactor.

**Figure 7:**
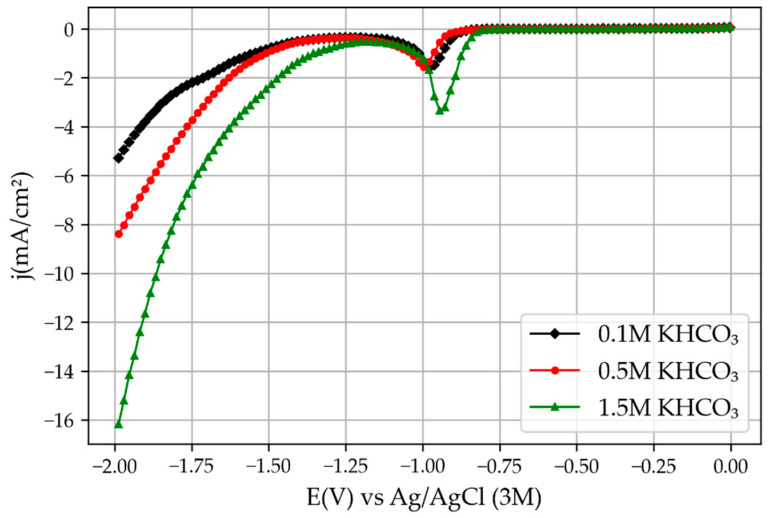
Linear voltammetry results obtained with the old CNC-manufactured module reprinted from [6].

The cyclic voltammetry results obtained by the novel 3D printed mini-reactor were comparable with the ones obtained with the CNC-manufactured reactor, but higher current densities were obtained, as one can see by comparing Figure 6 and Figure 7. In addition, the potential at which the peaks related to SnO_2_ reduction and the H_2_ evolution reaction appeared at lower potentials. The reason for this is the lower electric resistance between the two electrodes due to the reduced distance between them. For example, the novel 3D printed reactor was set with a distance between the two electrodes of around 7 mm, which is significantly smaller compared with the previously used reactor (46 mm). Moreover, the potential used for the chronoamperometry was 0.4 V lower than the potential used for the reactor from the previous study [6] for the same reasons: the lower the electrical resistance, the smaller the overpotential needed to trigger the reaction. This increase in the current density over the previous experiments with the bigger reactor also implies that the hydrogen evolution reaction would appear at lower overpotentials.

**Figure 8:**
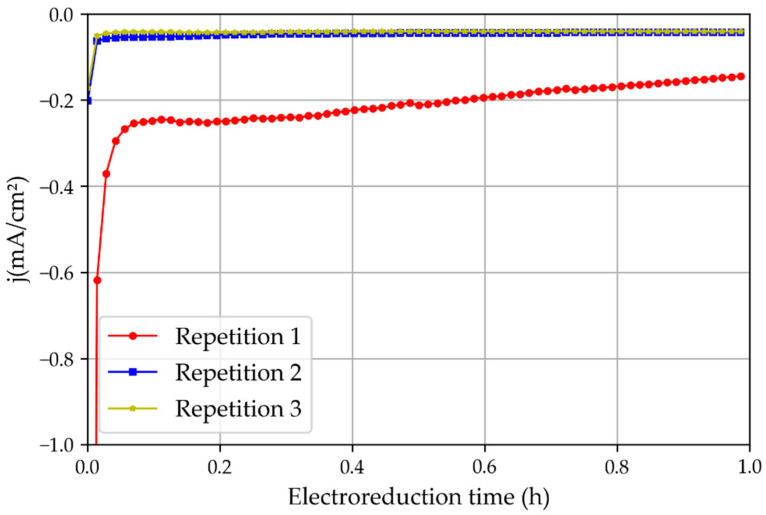
Three repetitions of the chronoamperometric test using a non-treated tin catalyst oxidated in the atmosphere and the 3D printed reactor.

The first set of electroreduction experiments was performed with tinfoil pre-oxidated in atmospheric air for 24 h. The chronoamperometric study showed that the first experiments had a significantly higher current density than the repetitions of the same experiment (Figure 8). This is because of the oxide layer generated on the tinfoil when it was exposed to air. This observation highlights the importance of the tin oxide layer for increasing the current density during CO_2_ electroreduction reactions [16].

**Figure 9:**
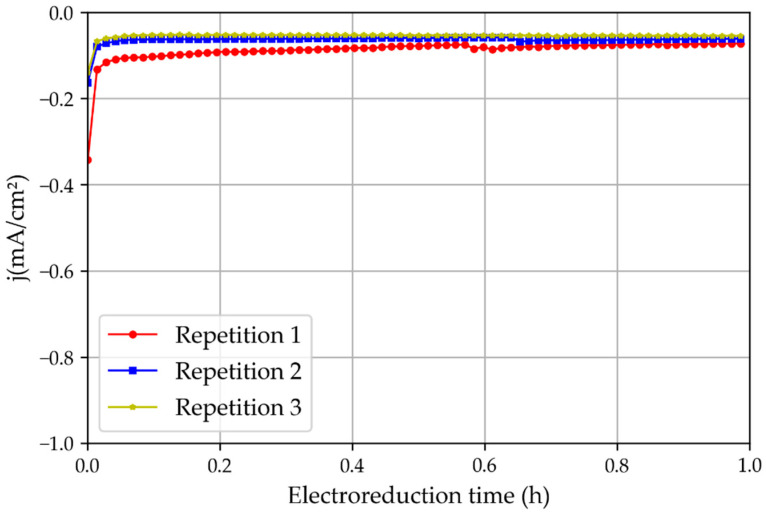
Three repetitions of the chronoamperometric test using new tin catalysts that had not been oxidated in the atmosphere and the 3D printed reactor.

To confirm the possibility that the higher current density obtained in Repetition 1 was caused by the oxide layer, another set of experiments was performed with new non-treated and non-pre-oxidized tinfoil. The results (Figure 9) showed that the first experiment presented only a slightly higher intensity than the later consecutive repetitions, possibly because of oxidation residues, but this was still significantly lower than those obtained when the foil was pre-oxidated for 24 h. Therefore, it can be stated that the native layer of tin oxide (SnO_2_) plays a very important role in the activity of the catalyst, as described in [17].

Furthermore, a quantification analysis of products was performed using NMR spectroscopy in order to determine if higher amounts/concentrations of formate could be obtained because of the higher current density with the 3D printed reactor. An NMR analysis of the liquid products was performed, and formate was observed only in the first repetition of the experiment, where the tin oxide layer was still not reduced. Therefore, this confirms the importance of the native oxide layer and demonstrates the need to test lower potentials in future experiments, to not reduce the native oxide layer and to maintain the activity of the catalyst over longer periods. The results obtained with the novel 3D printed membrane reactor cannot be directly compared with those obtained in the previous study, because of the reduction in the oxide layer, the different dimensions of the reactor and the different working potential used. Nonetheless, the faradaic efficiency was around 21%, which is extremely close to that obtained with previous rector (around 18%) when using the same concentration of bicarbonate. Another important point is that residual amounts of formate were found in the anodic compartment (below the quantification limits), proving the low crossover of formate towards anodic compartment and corroborating the good performance of the Nafion 117 proton exchange membrane in this application.

## 4. Conclusions

In general, 3D printing shows great capacity to substitute for classic CNC techniques for prototyping, mainly because of its low cost and fast manufacture, and the possibility of obtaining more complex parts, without the need to have a very expensive and specialized workshops and technicians. However, as said before, 3D printing is mainly for prototyping because the stability and the quality of the materials used is still relatively low. Therefore, most of the times when a final prototype has been achieved and is ready to be commercialized or used in large-scale production, classic workshop manufacture techniques show a great advantage. Thus, these techniques are complementary and each one has its own applications. 

In terms of the CO_2_ electroreduction performance, the 3D printed reactor showed greater capabilities due to the reduced distance between its electrodes, which decreased the electrical conductivity between them; therefore, the energy required was reduced together with a lower amount of the reagent needed for each experiment. The overall faradaic efficiency was not much greater with the 3D printed reactor, however. Moreover, it was confirmed that the oxide native layer present in tin (Sn) foil plays a very important role in CO_2_ electroreduction. A thicker proton exchange membrane might be needed in future studies in order to avoid crossover of the products.

Furthermore, this research will be expanded to automatic experimental systems using 3D technology, including the developed microreactor and the software to control it, in order to further accelerate the laboratory procedures and improve the repeatability of the results.

## Figures and Tables

**Table 1:** Comparation of the workshop-made reactor versus the 3D printed one.

Characteristics	Workshop Reactor	3D Printed Reactor
Reaction volume (mL)	30	1.5
Transparency	No	Yes
Cleaning	Hard	Easy
Tuning	Hard	Easy
Manufacturing time	Weeks	1 day
Manufacturing price (€)	500	5
Chemical tolerance	High	Low
Material variety	High	Low
Number of parts	More than 10	2
Number of bolts	30	4
Weight (g)	1780	47
Gastightness	Yes	No
Electrode–electrode Distance (mm)	46	7

## Data Availability

The data presented in this study are available on request from the corresponding author.
